# Family SES Is Associated with the Gut Microbiome in Infants and Children

**DOI:** 10.3390/microorganisms9081608

**Published:** 2021-07-28

**Authors:** Candace R. Lewis, Kevin S. Bonham, Shelley Hoeft McCann, Alexandra R. Volpe, Viren D’Sa, Marcus Naymik, Matt D. De Both, Matthew J. Huentelman, Kathryn Lemery-Chalfant, Sarah K. Highlander, Sean C. L. Deoni, Vanja Klepac-Ceraj

**Affiliations:** 1 Neurogenomics Division, Translational Genomics Research Institute (TGen), Phoenix, AZ 85004, USA; mnaymik@tgen.org (M.N.); mdeboth@tgen.org (M.D.D.B.); mhuentelman@tgen.org (M.J.H.); 2 Department of Biological Sciences, Wellesley College, Wellesley, MA 02481, USA; kbonham@wellesley.edu (K.S.B.); sm106@wellesley.edu (S.H.M.); 3 Advanced Baby Imaging Lab, Hasbro Children’s Hospital, Rhode Island Hospital, Providence, RI 02903, USA; lexievolpe@babyimaginglab.com (A.R.V.); viren_dsa@brown.edu (V.D.); sdeoni@mac.com (S.C.L.D.); 4 Department of Pediatrics, Warren Alpert Medical School, Brown University, Providence, RI 02912, USA; 5 Psychology Department, Arizona State University, Tempe, AZ 85281, USA; klemery@asu.edu; 6 Pathogen and Microbiome Division, Translational Genomics Research Institute North (TGen), Flagstaff, AZ 86005, USA; shighlander@tgen.org; 7 MNCH D&T, Bill and Melinda Gates Foundation, Seattle, WA 98109, USA

**Keywords:** socioeconomic status, infant, childhood, microbiome, stress

## Abstract

Background: While early life exposures such as mode of birth, breastfeeding, and antibiotic use are established regulators of microbiome composition in early childhood, recent research suggests that the social environment may also exert influence. Two recent studies in adults demonstrated associations between socioeconomic factors and microbiome composition. This study expands on this prior work by examining the association between family socioeconomic status (SES) and host genetics with microbiome composition in infants and children. Methods: Family SES was used to predict a latent variable representing six genera abundances generated from whole-genome shotgun sequencing. A polygenic score derived from a microbiome genome-wide association study was included to control for potential genetic associations. Associations between family SES and microbiome diversity were assessed. Results: *Anaerostipes*, *Bacteroides*, *Eubacterium*, *Faecalibacterium*, and *Lachnospiraceae* spp. significantly loaded onto a latent factor, which was significantly predicted by SES (*p* < 0.05) but not the polygenic score (*p* > 0.05). Our results indicate that SES did not predict alpha diversity but did predict beta diversity (*p* < 0.001). Conclusions: Our results demonstrate that modifiable environmental factors influence gut microbiome composition at an early age. These results are important as our understanding of gut microbiome influences on health continue to expand.

## 1. Introduction

The human gut microbiota play an important role in a broad range of physiological functions, including immune system maturation, metabolic and inflammatory processes, and the central nervous system [1,2,3]. Microbial diversity is influenced by a combination of environmental and host genetic factors and is associated with several complex diseases [4]. Various environmental factors influence inter-individual variation in the gut microbiota structure and function during childhood, including mode of birth (vaginal or caesarean section), initiation and duration of breastfeeding, antibiotic exposure, indoor and outdoor environment, pet exposure, and diet [5,6].

While the human gut microbiome composition is strongly shaped by various environmental factors, there is also evidence for host genetic influence [7]. Early underpowered studies suggested that monozygotic twins were no more similar in gut microbiota than dizygotic twins, suggesting very little, if any, genetic influence [8,9]. However, more recent studies incorporating larger twin samples from the TwinsUK cohort show a small but statistically significant effect of genetics on microbiome composition [10,11]. Studies in mice further demonstrate the role of host genetic influence on microbiome composition [12,13,14]. Importantly, molecular genetic studies [15] and multiple genome-wide association studies (GWAS) have demonstrated that host genetics moderately influence microbial composition in humans [16,17,18,19,20,21]. Together, these studies demonstrate the existence of human genes with alleles that contribute to microbiome composition.

As our understanding of environmental and genetic influences on gut microbiome composition continue to grow, recent research suggests the social environment may also play a role in regulating the gut microbiome [22]. Research linking the social environment to the gut microbiome mostly stems from animal models focused on the impact of social interactions and psychosocial stress. Studies in primates suggest that social relationships influence gut microbiome composition through microbial sharing between individuals [23,24,25,26]. In addition to direct microbial sharing, psychosocial stressors may also affect microbiome structure [27,28]. Animal studies demonstrate that psychological stressors, such as novel housing, social disruption, restraint stress, and early life stress, affect microbial community compositions [29,30,31]. Prenatal maternal stress and interruption of maternal care have also been shown to impact the gut microbiota of offspring in mice [25,30,32,33,34]. Collectively, this body of evidence suggests an association between social factors and the microbiome, encouraging studies of these relations in humans.

Research to date on social factors and the microbiome in humans is limited. Socioeconomic status (SES) is a factor that reflects economic resources such as education, income, and occupation. As SES is related to living conditions, psychosocial stress, and nutrition, it is likely that SES plays a major role in influencing the gut microbiome [35,36]. A study of forty-four healthy adults found that lower neighborhood-level SES in Chicago, Illinois was associated with reduced alpha-diversity, greater abundance of taxa associated with the genus *Bacteroides*, and lower abundance of taxa associated with the genus *Prevotella* in the gut microbiota [37]. Bowyer et al. (2019) extended this work using a large sample of adult twins in the United Kingdom. This study found associations between individual and area-level income and relative abundance of operational taxonomic units (OTUs) in the gut microbiome [38]. They also found individual and area-level incomes were linked to microbial composition and lower alpha diversity [38]. However, much less research has investigated the relationships between SES and gut microbiome composition in children.

These two studies are an important first step in describing associations of SES and the microbiome. The current study extends this work by testing the associations between family SES with relative abundance of genera and diversity in the gut microbiome of infants and children while controlling for potential genetic associations. We use genera previously associated with SES in adults [38] in a structural equation model framework. Genetic associations were controlled for with polygenetic scores (PGS) generated from the most recent and largest microbiome GWAS findings [20]. Although cross-sectional data cannot be used to demonstrate causality, structural models yield fit indices that represent the fit of the data to the hypothesized model.

## 2. Materials and Methods

### 2.1. Demographics and Family SES

Demographic and socioeconomic characteristics (child age, child sex, parental education, and birth type) were collected by parental-report. Family SES was measured by averaging paternal and maternal education [39]. Education levels were coded as follows: Less Than Seventh Grade = 1; Junior High School = 2; Partial High School = 3; High School Graduate = 4; Partial College or Specialized Training = 5; College Graduate = 6; Graduate Training = 7. Written consent was obtained from parents or legal guardians in accordance with ethics approval from the host institution’s Institutional Review Board.

### 2.2. SNP Microarray and Polygenic Scores (PGS)

Saliva was collected from participants in the lab using Oragene (DNA Genotek, Ottawa, ON, Canada) saliva collection kits. DNA was extracted with a standard isolation kit (DNA Genotek’s PT-L2P-5). Sample yield and purity were assessed spectrophotometrically using NanoDrop ND-1000 (ThermoScientific, Wilmington, DE, USA) methods. Genotyping of the Multi-Ethnic Global Array (MEGA, >1.7 million markers) run on an Illumina iScanSystem (Illumina, San Diego, CA, USA) was conducted at the Translational Genomics Research Institution (TGen, Phoenix, AZ, USA). Initial genotype definitions were based on auto-clustering all samples that had call rate >0.98 using the GenomeStudio software (2.0). Following genotype calling on all samples, VCF files were imported to the TOPMed Imputation Server [40] and built with the hg38 reference for whole genome imputation followed by a liftover to build hg19 for PGS.

PGS were generated according to standardized, published methods [41]. In summary, using the ‘’P/A’’ variants discovered by Hughes et al. (2020), the count of each effect allele was multiplied by the natural log of the odds ratio for that allele and then summed across all SNPs for each individual. The sum of all allelic effects represents the PGS.

### 2.3. Stool Sample Collection and Handling

Stool samples were collected by parents in OMR-200 tubes (OMNIgene GUT, DNA Genotek, Ottawa, Ontario, CA, USA), stored on ice, and brought within 24 h to the laboratory in RI where they were immediately frozen at −80 °C. Stool samples were not collected if the infant had taken antibiotics within the last two weeks. Samples were transported to Wellesley College (Wellesley, MA, USA) on dry ice for further processing.

### 2.4. DNA Extraction and Sequencing of Metagenomes

Nucleic acids were extracted from 200 µL of each stool sample using the RNeasy PowerMicrobiome kit automated on the QIAcube (Qiagen, Germantown, MD, USA), excluding the DNA degradation steps. Cell lysing steps were performed using the Qiagen PowerLyzer 24 Homogenizer (Qiagen, Germantown, MD, USA) at 2500 speed for 45 s, then samples were transferred to the QIAcube to complete the protocol, and extracted DNA was eluted in a final volume of 100 µL. Extracted DNA was sequenced at the Integrated Microbiome Resource (IMR, Dalhousie University, NS, Canada) [42]. To sequence metagenomes, a pooled library (max. 96 samples per run) was prepared using the Illumina Nextera Flex Kit for MiSeq and NextSeq (a PCR-based library preparation procedure) from 1 ng of each sample where samples were enzymatically sheared and tagged with adaptors, PCR amplified while adding barcodes, purified using columns or beads, and normalized either using Illumina beads or manually. Samples were then pooled onto a plate and sequenced on the Illumina NextSeq 550 platform using 150 + 150 bp paired-end “high output” chemistry, generating ~400 million raw reads and ~120 Gb of sequence. The average sequencing depth per sample was 7,738,479. Samples were deposited in NCBI Genbank under Bioproject PRJNA695570.

### 2.5. Analyzing Metagenomes

**Figure 1:**
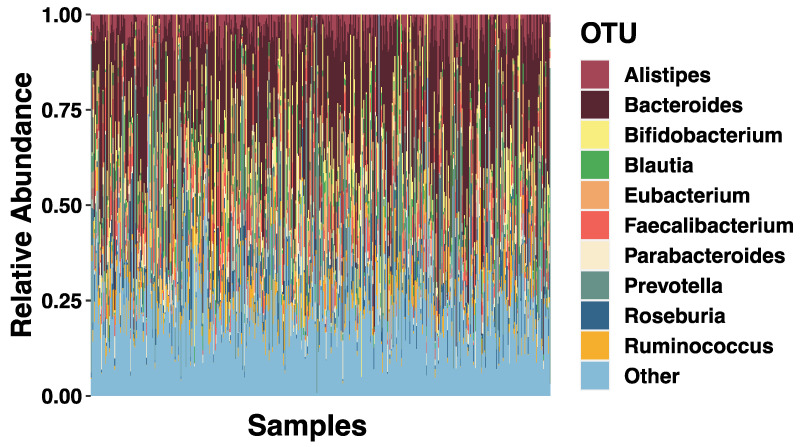
Full Sample Taxonomic Summaries. Stacked bar plots showing the average relative abundance of the top ten. Different colored bars represent different genus (indicated by the key). Any other genus are classified as ‘’Other’’.

Metagenomic data were analyzed using bioBakery workflows with all necessary dependencies and default parameters [43]. Briefly, KneadData (v0.7.10) was used to trim and filter raw sequence reads, and to separate human and 16S ribosomal gene reads from bacterial sequences in fecal samples. Samples that passed quality control were taxonomically profiled to the genus level using MetaPhlAn (v3.0.7) [44]. Full taxonomic breakdown at the genus level showing top ten microorganisms can be found in Figure 1.

### 2.6. Structural Equation Modeling

Using the lavaan [45] package in R [46], a latent variable path model within the structural equation modeling (SEM) framework was used to investigate relationships between family SES and the gut microbiome in infants and young children. SEM is a multivariate method where complex relationships between exposures and outcome variables can be estimated simultaneously in a single model, with error measured separately. A latent variable path model is a statistical model of hypothesized relationships among a set of latent and observed variables. In statistics, latent variables are variables that are not directly observed but are rather inferred from other directly measured variables. Latent variables represent “shared” variance, or the degree to which variables “move” together, therefore, variables that have no correlation cannot result in a latent construct. The use of latent variables can serve to reduce the dimensionality of data since many observable variables can be aggregated into a single latent variable.

**Figure 2:**
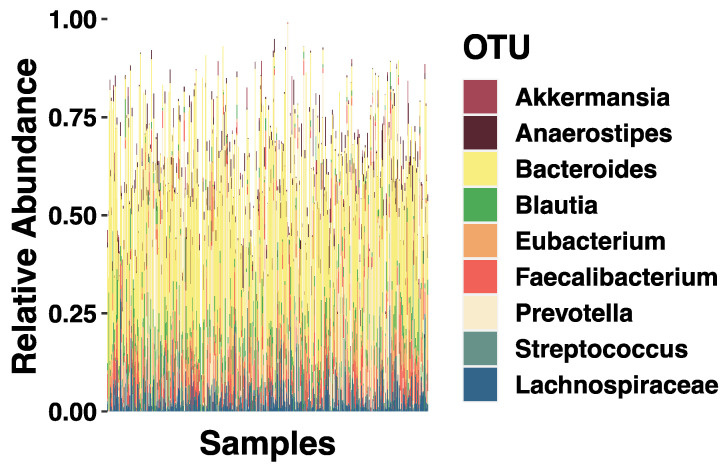
SEM Model Taxonomic Summaries. Stacked bar plots showing the average relative abundance of the genera assessed with socioeconomic status (SES). Different colored bars represent different genus (indicated by the key).

The underlying theoretical framework includes assumptions about the causality of relationships suggested by the literature such that early environmental exposures influence microbiome structure. The latent variable SEM can easily incorporate covariates such as sex, age, birth type, and sequencing depth to examine their influences on the underlying microbiome composition. Relationships are estimated by solving regression-style equations based on a variance-covariance matrix and all correlations between variables are modeled [47]. This is an advantage in handling microbiota relative abundance data since profiles derived from sequencing data are inherently relative and correlated [48,49]. While SEM is still a relatively novel statistical approach with microbiome data, others have had success with it [50,51]. Taxonomic breakdown of the genera assessed in this study can be found in Figure 2.

Participant data were chosen based on available microbiome data. For SEM, all other variables were subjected to full information maximum likelihood (FIML) estimation, which has been shown to be superior compared to listwise deletion, pairwise deletion, and similar response pattern imputation [52]. The basic premise of FIML is that instead of imputing the values of missing data, we estimate missing values by determining the value that maximizes the likelihood function based on the sample data that we have.

Model goodness of fit (GOF) was evaluated jointly by two commonly used fit indices, root mean square error of approximation (RMSEA) and the standardized root mean square residual (SRMR). GOF was considered acceptable if the RMSEA and SRMR were less than 0.08 [53,54].

### 2.7. Microbiome Diversity

Alpha diversity measure (Shannon index) was calculated for each individual using the R package vegan. Bray–Curtis dissimilarities, the distances to group centroids, were used as measures of beta diversity, using the function vegdist within vegan. Permutational multivariate analysis of variance (PERMANOVA) was performed with the family SES variable (9999 permutations) within vegan to test associations.

## 3. Results

### 3.1. Sample Characteristics

A summary of sample descriptive statistics can be found in Table 1.

### 3.2. SES and Relative Abundance

The primary aim of the present study was to examine the relations between SES with childhood microbiome abundance and diversity. To test these predictions, we used the lavaan package in R, a structural equation modeling program. As a first step in our analyses, we tested a measurement model with one latent variable, indicated by the relative abundance of the genera *Akkermansia*, *Anaerostipes*, *Bacteroides*, *Blautia*, *Eubacterium*, *Faecalibacterium*, *Lachnospiraceae*, *Prevotella*, and *Streptococcus.* The initial model was based on the predefined theoretical framework and then trimmed by dropping indicators that were not statistically significant, specifically *Akkermansia*, *Prevotella*, *Streptococcus*, and *Blautia* from the model to improve model fit. The revised model fit the data well (RMSEA = 0.068, SRMR = 0.036), was a significant improvement from the first model (χ2Δ[22] = 332.43, *p* < 0.00001), and provided a basis for further model testing. All loadings were significant (all *p*’s < 0.01).

**Figure 3:**
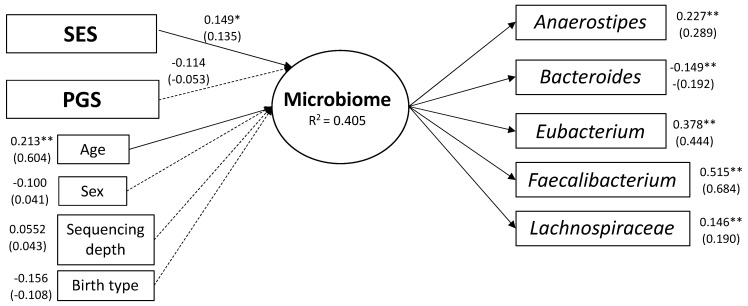
Latent Variable Path Model-Socioeconomic Status (SES) and Microbiome. Gut microbiome genera previously associated with SES in adulthood load onto a single latent variable which is predicted by SES and age in young children. The shapes in the graph represent variables where squares are observed variables and circles are latent variables. The single headed arrows are regression effects, solid lines indicates a significant path and dashed line indicates non-significance. Unstandardized estimates are above the standardized estimates, which are in parentheses. * *p* < 0.05, ** *p* < 0.001. RMSEA = 0.062; SRMR = 0.047.

Next, we added SES, PGS, child sex, child age, birth type, and sequencing depth as predictors of the microbiome latent variable. The hypothesized model fit the data well (CFI = 0.811; RMSEA = 0.062, SRMR = 0.047; Figure 1). SES was a significant predictor of the latent microbiome factor (β = 0.135, *p* < 0.05). PGS was not a significant predictor of the microbiome latent factor (*p* > 0.05). Of all the covariates, only child age was a significant predictor (β = 0.604, *p* < 0.001). The full model and results can be found in Figure 3.

### 3.3. Alpha Diversity

Using a linear regression model, we tested if Shannon index (alpha diversity) was predicted by SES with the covariates of child’s age, child’s sex, birth type, and sequencing depth. SES was not a significant predictor of the Shannon index (*p* > 0.05). Of all the covariates, as expected, only child age was a significant predictor of alpha diversity ((β = 0.061, *p* < 0.001).

### 3.4. Beta Diversity

**Figure 4:**
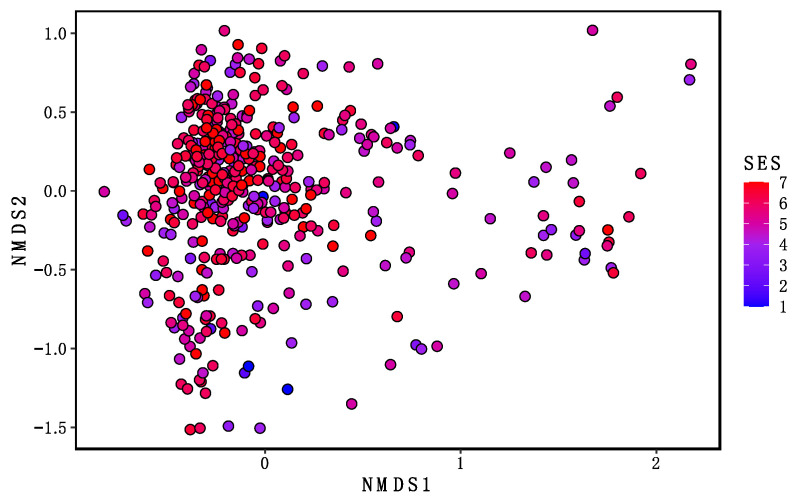
Socioeconomic Status (SES) and Beta Diversity. Beta diversity was calculated based on Bray-Curtis Dissimilarity Distances and visualized using a Non-metric Multidimensional Scaling (NMDS) plot. The NMDS plot projects dissimilarity onto a plane with the two axes representing the largest variance. Data points represent individual beta-diversity values and have been colored by SES score to visualize the relationship between SES and beta-diversity. F(1, 432) = 8.46, *p* = 0.0001; R2 = 0.0192; Stress value = 0.17.

Examining intra-individual (beta) microbiome diversity, we found significant differences across SES for Bray–Curtis dissimilarity (*F*(1, 432) = 8.46, *p* = 0.0001; R^2^ = 0.0192; Figure 4). This suggests a difference in microbiota community composition across SES levels.

## 4. Discussion

Although studies have investigated the relation between SES and gut microbiome in adults and many studies have investigated different lifestyle factors and the gut microbiome in early infancy [5,6,55,56,57], knowledge on the associations between SES and gut microbiome in young children is lacking. This study focused on evaluating the associations between family SES and child genetics with the bacterial community structure of the gut microbiome in a cohort of infants and children. Using SEM, we found similar relationships between SES and composition of the gut microbiome at the genera level to the composition reported in previous studies of adults [37,38].

Using genera associated with SES in adulthood, we tested the association between nine microorganisms and SES in a latent variable path model. We found *Anaerostipes*, *Bacteroides*, *Eubacterium*, *Faecalibacterium*, and *Lachnospiraceae* all significantly load onto a single latent factor that was predicted by SES and by child’s age. The average relation across our sample between SES and microbiome was 0.15 (Figure 3). Parents with higher years of education had children who scored higher on the latent microbiome factor. That is, they were higher on *Faecalibacterium*, *Eubacterium*, *Anaerostipes*, and *Lachnospiraceae*, and lower on *Bacteroides*. These results align well with previous studies that found associations with adult individual—or area—SES with relative abundance of these genera.

Of note, the genera *Faecalibacterium* had the highest factor loading (0.68). The genus *Faecalibacterium* belongs to the family of *Ruminococcaceae. F. prausnitzii*, a species belonging to this genera, is considered a key marker for a healthy gut and has the ability to produce butyrate, a short chain fatty acid, by consuming acetate [58,59]. Butyrate is an energy source for the colonic epithelium and plays a major role in gut physiology and has several beneficial effects for health including protection against pathogens, modulation of immune system, and reduction of cancer progression [60]. One study in six-year-olds found non-smoking mothers, vaginal birth, and high family SES were all associated with increased relative abundance of *Faecalibacterium* [61]. Together, with the findings of this study, this suggests that *Faecalibacterium* abundance may be susceptible to a wide range of environmental factors. Therefore, *Faecalibacterium* abundance may be one biological pathway in which early environmental influences shape disease vulnerability through life.

Alpha diversity captures the diversity of species, or the evenness and richness of microbial composition [62]. Reduced alpha diversity has been associated with brain-based disorders in adulthood such as Alzheimer’s disease and major depressive disorder, and in children with attention-deficit/hyperactivity disorder (ADHD) [63,64,65]. We found that alpha diversity increases with age, as has been shown in other studies [66,67]. However, our results showed no association between family SES and the overall composition of the gut microbiome in infants and young children (alpha diversity). This is consistent with another study that reported no association between family SES and alpha diversity in six-year-olds [61]. These findings depart from two studies in adults that found family and neighborhood-level SES were associated with alpha diversity in adults [37,38]. Interestingly, this pattern of findings suggests that microbiome alpha diversity may not be sensitive to the impact of SES until a later developmental stage. However, we did find that SES significantly predicts beta-diversity, as measured by Bray–Curtis dissimilarity, suggesting gut microbiome diversity is influenced by family SES in childhood to some degree. These findings may have important implications for understanding how interventions in childhood could help prevent the eventual impact of SES on microbiome diversity and subsequent health.

Interest in the interplay between host genetics and the gut microbiome is increasing, with many GWAS studies to date [16,17,18,19,20,21]. Polygenic scores are calculated by summing the number of risk alleles, which are weighted by effect sizes derived from GWAS results [41]. Polygenic studies have demonstrated modest prediction for many complex phenotypes including blood pressure [68,69], height [70], diabetes [71], obesity [72], ADHD [73], depression [74], and schizophrenia [75]. A recent study found the polygenic score for arthritis was associated with the presence of *Prevotella* spp. in the microbiome [76]. Using the most recent and largest-to-date microbiome GWAS study [20], we calculated individual PGSs. We did not find an association between the PGS and microbiome composition in children, suggesting that SES may have more impact than genetics at this age. However, there are multiple explanations for this null finding. One of the major reasons may be the limited number of microorganisms assessed in this study, perhaps the PGS may be associated with genera not included here. Another interesting possibility is that the genetic influence on microbiome composition may have a specific developmental window. In other words, a PGS based on a GWAS conducted in adults may not be associated with microbiome composition in childhood. In addition, other factors such as ethnic differences between studies, gene-environment interactions, and dissimilarity in sequencing methods, might also make it difficult to detect a genetic association with microbiome composition in this study.

There are some limitations of the current study to address. As we only had two indicators of family SES, we used a manifest composite variable rather than a latent factor in the model. Use of a manifest variable representing family SES likely underestimated the association with microbiome, as it included measurement error. Using maternal and paternal education as a measure of SES may have imperfect representation of this complex variable. Future studies should consider incorporating other indicators of family SES such as income, occupation, and area-level metrics. Further, we did not have access to diet variables such as food quantity/quality or diversity, which could be driving the associations observed with SES. Since SES is associated with a variety of lifestyle factors such as medication use, pet ownership [5], psychosocial stress [77], and host environment [9,78], we are unable to determine which of these factors is driving associations with microbiome metrics. Associations found with SES in this study were small in magnitude. This may be due to the restricted SES range in our volunteer longitudinal cohort which are known to be biased towards higher SES households. While detailed information on the history of antibiotic use is missing in this study, we did exclude any participants who reported antibiotic use within two weeks prior to collection.

## 5. Conclusions

Our results demonstrate that modifiable environmental factors, such as SES, may influence gut microbiome composition at an early age. Further, our results suggest that host genetics are not associated with the taxa tested in early life. These results are important as the understanding of gut microbiome-host health relationships continue to expand. Our future research will explore if children’s microbiome mediates the well-established relationships between SES and children’s academic and health functioning.

## Figures and Tables

**Table 1:** Sample descriptive statistics.

Variable	N	Mean (SD) or %	Range
Metagenomics	588	-	-
Age (years)	315	4.5 (3.63)	1 m–15 y
Sex (Female)	547	45%	-
Socioeconomic status (SES)	434	4.2 (1.87)	1–7
PGS	358	0.36 (1.42)	−2.72–2.79
Birth type	370	69% (Vaginal)	-
Race	406	60.6% White; 26.8% Mixed; 7.6% African American; 1.2% Asian; 1.2% Native American; 2.5% Declined	-
--Alpha-Diversity (Shannon)	588	2.05 (0.56)	0.09–3.02

## Data Availability

Samples were deposited in NCBI Genbank under Bioproject PRJNA695570.
